# Protein disulphide isomerase can predict the clinical prognostic value and contribute to malignant progression in gliomas

**DOI:** 10.1111/jcmm.15264

**Published:** 2020-04-17

**Authors:** Qing Hu, Kai Huang, Chuming Tao, Xingen Zhu

**Affiliations:** ^1^ Department of Neurosurgery The Second Affiliated Hospital of Nanchang University Nanchang China; ^2^ East China Institute of Digital Medical Engineering Shangrao China

**Keywords:** glioma, prognostic signature, protein disulphide isomerase

## Abstract

Increasing evidence from structural and functional studies has indicated that protein disulphide isomerase (PDI) has a critical role in the proliferation, survival and metastasis of several types of cancer. However, the molecular mechanisms through which PDI contributes to glioma remain unclear. Here, we aimed to investigate whether the differential expression of 17 PDI family members was closely related to the different clinicopathological features in gliomas from The Cancer Genome Atlas (TCGA) and Chinese Glioma Genome Atlas data sets. Additionally, four subgroups of gliomas (cluster 1/2/3/4) were identified based on consensus clustering of the PDI gene family. These findings not only demonstrated that a poorer prognosis, higher WHO grade, lower frequency of isocitrate dehydrogenase mutation and higher 1p/19q non‐codeletion status were significantly correlated with cluster 4 compared with the other clusters, but also indicated that the malignant progression of glioma was closely correlated with the expression of PDI family members. Moreover, we also constructed an independent prognostic marker that can predict the clinicopathological features of gliomas. Overall, the results indicated that PDI family members may serve as possible diagnostic markers in gliomas.

## 1 INTRODUCTION

The protein disulphide isomerase (PDI) family of endoplasmic reticulum (ER)‐resident enzymes is responsible for the formation, cleavage and rearrangement of protein disulphide bonds.^1^ The primary function of PDIs is the formation of S–S bonds through a thiol‐disulphide exchange reaction, which is essential for the maturation and stability of most secreted and membrane‐localized proteins.^2^, ^3^, ^4^ The PDI family of proteins belongs to the thioredoxin (TRX) superfamily and comprises 21 members. Although their length, domain arrangement and substrate specificity are different, they share a common TRX‐like domain.^3^, ^5^, ^6^ Analysis of gene expression microarray data sets has revealed that, when compared with normal tissues, the expression of PDI is increased to varying degrees in different cancer types,^1^ such as those of the brain and CNS,^7^ lung,^8^ kidney ^9^ and ovary.^10^

Extensive research over the past few decades has strongly suggested that PDI is significantly associated with cancer progression. For example, PDI protein expression was significantly increased in axillary lymph node metastatic breast tumours when compared with that in primary breast tumours.^11^ Using global proteomics methods, studies have shown that PDI is strongly expressed in invasive glioma cells, in both human xenografts and invasive glioma fronts.^12^ Inhibition of PDIA1 activity was shown to increase the apoptosis of melanoma cells and suppress tumour growth in human ovarian cancer mouse xenografts.^13^, ^14^ Moreover, down‐regulation of PDIA6 was reported to inhibit the proliferation and invasion of bladder cancer cells.^15^ Additionally, knockdown of PDIA4 was indicated to contribute to a reduction in hepatic tumorigenesis and increase the survival of mice with spontaneous hepatoma,^16^ while PDI was also reported to be closely correlated with the resistance of HeLa cells to the chemotherapeutic agent plitidepsin.^17^ This suggests that the expression levels of PDI family members in cancer can potentially be used as prognostic markers in glioma. However, no comprehensive investigation has been undertaken on the function and prognostic value of PDIs in the malignant glioma progression.

Here, we compared the expression of 17 well‐characterized PDI family members with the clinicopathological characteristics of 689 glioma samples obtained from The Cancer Genome Atlas (TCGA) data portal and 508 glioma samples downloaded from the Chinese Glioma Genome Atlas (CGGA) data portal. The prognostic value of PDI family members in glioma was also evaluated based on 9 PDI members selected by least absolute shrinkage and selection operator (LASSO) Cox regression analysis.

## 2 MATERIALS AND METHODS

### 2.1 Data sets for glioma samples

The RNA‐seq data and corresponding clinicopathological information of 689 and 508 glioma samples were downloaded from TCGA (http://cancergenome.nih.gov/) and CGGA (http://www.cgga.org.cn/), respectively. The somatic mutation profiles were downloaded from the Genomic Data Commons data portal (https://portal.gdc.cancer.gov/) and analysed by the R package ‘maftools’. All of the RNA‐seq data were normalized by log 2(n + 1) transformation. The clinicopathological information for the CGGA and TCGA data sets is summarized in Table S1.

### 2.2 Selection of PDI family members

A total of seventeen PDI members were obtained by combining the two selection methods, namely the selection of 21 PDI family members based on the published literature ^1^, ^18^ and the RNA expression data available in TCGA and CGGA data sets.

### 2.3 Oncomine and human protein atlas databases

The ONCOMINE database (http://www.oncomine.org) is an integrated online cancer microarray database based on DNA or RNA sequence analysis, and a convenient tool for the analysis of genome‐wide gene expression. According to the ONCOMINE database, the mRNA expression levels of 17 PDI family members in different cancer samples were compared to their corresponding normal control samples. Differences in expression levels were analysed by Student's *t* test. Threshold values were set as follows: *P*‐value: 1 × 10^−4^; fold change: 2; gene rank; 10%; and data type: mRNA. Furthermore, the Human Protein Atlas (https://www.proteinatlas.org/) was utilized to validate the protein levels of the 17 PDIs.

### 2.4 Bioinformatic analysis

To investigate the function of PDI family members in glioma, the R package ‘limma’ was used to correlate the expression of the 17 PDIs with the different clinicopathological features of glioma patients. The correlation between PDI family members was analysed using Spearman's correlation test. Next, 689 glioma samples in TCGA data were grouped using the R package ‘ConsensusClusterPlus’, and principal component analysis (PCA) was used to verify the grouping results. The Kaplan‐Meier method was used for survival analysis. In addition, GO and KEGG analyses were conducted for genes with differential expression between the C1 (cluster 1/2/3) and C2 (cluster 4) clusters using the Database for Annotation, Visualization, and Integrated Discovery (DAVID, http://david.abcc.ncifcrf.gov/home.jsp). Gene set enrichment analysis (GSEA) was also performed to validate the functions of genes differentially expressed between C1 and C2.

To determine the prognostic value of PDI family members, univariate Cox regression analysis of their expression in TCGA data set was performed. From this, we identified nine genes significantly associated with survival (*P* < .05), and these genes were selected for further functional analysis and development of a potential risk signature using the LASSO Cox regression algorithm. Subsequently, the nine genes and their coefficients were selected by the optimal value for penalization coefficient lambda, which was analysed by the smallest 10‐fold cross‐validation within the training (TCGA) data set. The measure of survival risk was calculated by the risk score for each patient in both the training (TCGA) and validation (CGGA) data sets using the following formula: Risk score = expression of gene 1 × *β*1 + expression of gene 2 × *β*2 + … expression of gene *n* × *βn*. The median cut‐off value for the prognostic risk score was utilized to categorize patients into low‐ and high‐risk subgroups. A time‐dependent receiver operating characteristic (ROC) curve analysis within 3 and 5 years was used to evaluate the predictive accuracy and sensitivity of our prognostic model. Tumour mutation burden (TMB) was calculated using Perl scripts (https://www.perl.org/), and the algorithm to calculate the TMB was the total number of somatic mutations (including non‐synonymous point mutations, insertions and deletions in the coding region)/size of the target region, in units of mutations/Mb.

### 2.5 Statistical analysis

One‐way ANOVA was used to compare the expression levels of PDI family members and TMBs in gliomas of different WHO grades. The expression levels of PDI family members according to isocitrate dehydrogenase (IDH) status and 1p/19q codeletion status in gliomas were analysed by *t* tests, which were also used to compare the association between TMB and risk score in somatic mutation profiles of gliomas. Chi‐squared tests were used to compare the distribution of gender, WHO grade, TCGA subtype, IDH status and 1p/19q codeletion status between the low‐ and high‐risk groups using the median risk score (derived from the risk signature) as the cut‐off value. The prognostic value of the risk score and various clinical and molecular‐pathological characteristics were compared by univariate and multivariate Cox regression analyses. Receiver operating characteristic (ROC) curves were generated to test the prediction efficiency of the risk signature, WHO grade, and age for 3‐ and 5‐year survival. The overall survival (OS) of the patients in the four subgroups of gliomas (cluster 1/2/3/4), low‐ and high‐risk groups, low‐ and high‐TMB groups, or different WHO grades of glioma based on the, respectively, median risk score were compared by the Kaplan‐Meier method. GraphPad Prism 7 (GraphPad Software, Inc), R v3.4.1 (https://www.r-project.org/) and SPSS 16.0 (SPSS Inc) were used to conduct the statistical analyses. *P* < .05 was considered significant.

## 3 RESULTS

### 3.1 Expression of PDI family members was significantly associated with clinicopathological features in glioma

**Figure 1:**
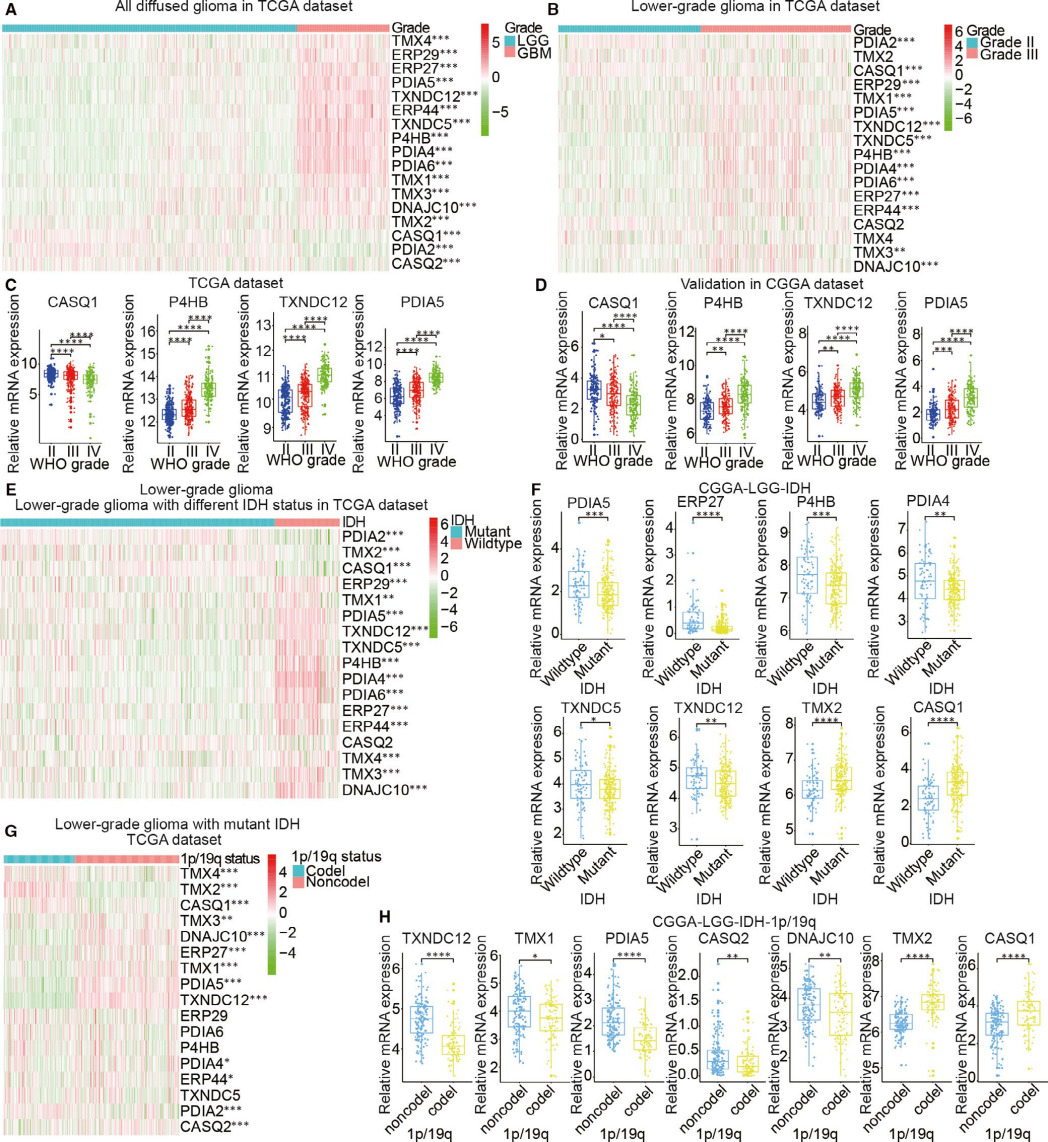
Expression of protein disulphide isomerase (PDI) in gliomas with different clinicopathological features. (A–D) Expression levels of PDI family members in gliomas of different WHO grades. (E, F) Expression levels of PDI family members in low‐grade gliomas (LGG) with differing isocitrate dehydrogenase (IDH) status. (G, H) Expression levels of PDI family members in IDH‐mutant LGGs with differing 1p/19q codeletion status

Accumulating evidence has indicated that the PDI enzyme family is actively involved in the occurrence and development of various cancers. To clarify the biological function of PDI members in glioma occurrence and development, we comprehensively analysed the relationship between 17 PDI family members and glioma based on different clinicopathological features, such as WHO grade, IDH status and 1p/19q codeletion status. The 689 glioma samples obtained from TCGA were used as the training set, and the 508 glioma samples obtained from the CGGA were used for verification (Table S1). A heat map was generated to visualize the correlation between the mRNA levels of the 17 PDI members and glioma WHO grades based on TCGA data set (Figure 1A,B), with the results indicating that most of the 17 PDIs were significantly correlated with glioma WHO grade. Furthermore, quantitative analysis of the mRNA levels from both data sets showed that, compared with those in low‐grade glioma (LGG) samples (WHO grade II and III), the expression levels of *P4HB*, *TXNDC12* and *PDIA5* were significantly up‐regulated in glioblastoma multiforme (GBM) samples (WHO grade IV), whereas that of *CASQ1* were down‐regulated (Figure 1C,D). Additionally, the 17 PDIs were closely related to the IDH and 1p/19q status. Here, we only considered the IDH status of LGG samples as only 11 IDH‐mutant high‐grade glioma (HGG) samples were identified. As shown in Figure 1E, most of the 17 PDIs were correlated with IDH status. We also investigated the relationship between the 17 members and 1p/19q status in IDH‐mutant LGG samples (Figure 1G). The results showed that 13 of the PDIs were significantly correlated with 1p/19q status. The genes validated in the CGGA data set presented an expression pattern consistent with that of TCGA data set, that is the expression levels of *PDIA5*, *ERP27*, *P4HB*, *PDIA4*, *TXNDC5* and *TXNDC12* were higher in the LGG samples with wild‐type IDH than in those with mutated IDH. In contrast, the levels of *TMX2* and *CASQ1* were significantly up‐regulated in the mutant IDH state compared with that in the wild‐type state (Figure 1F). In the IDH‐mutant LGG samples of the CGGA data set, the mRNA levels of *TXNDC12*, *TMX1*, *PDIA5*, *CASQ2* and *DNACJ10* were significantly higher in the LGG samples without 1p/19q codeletion, while those of *TMX2* and *CASQ1* were increased in LGG samples presenting with 1p/19q codeletion (Figure 1H).

### 3.2 Identification of PDI family members as potential biomarkers based on oncomine and human protein atlas database analyses

**Figure 2:**
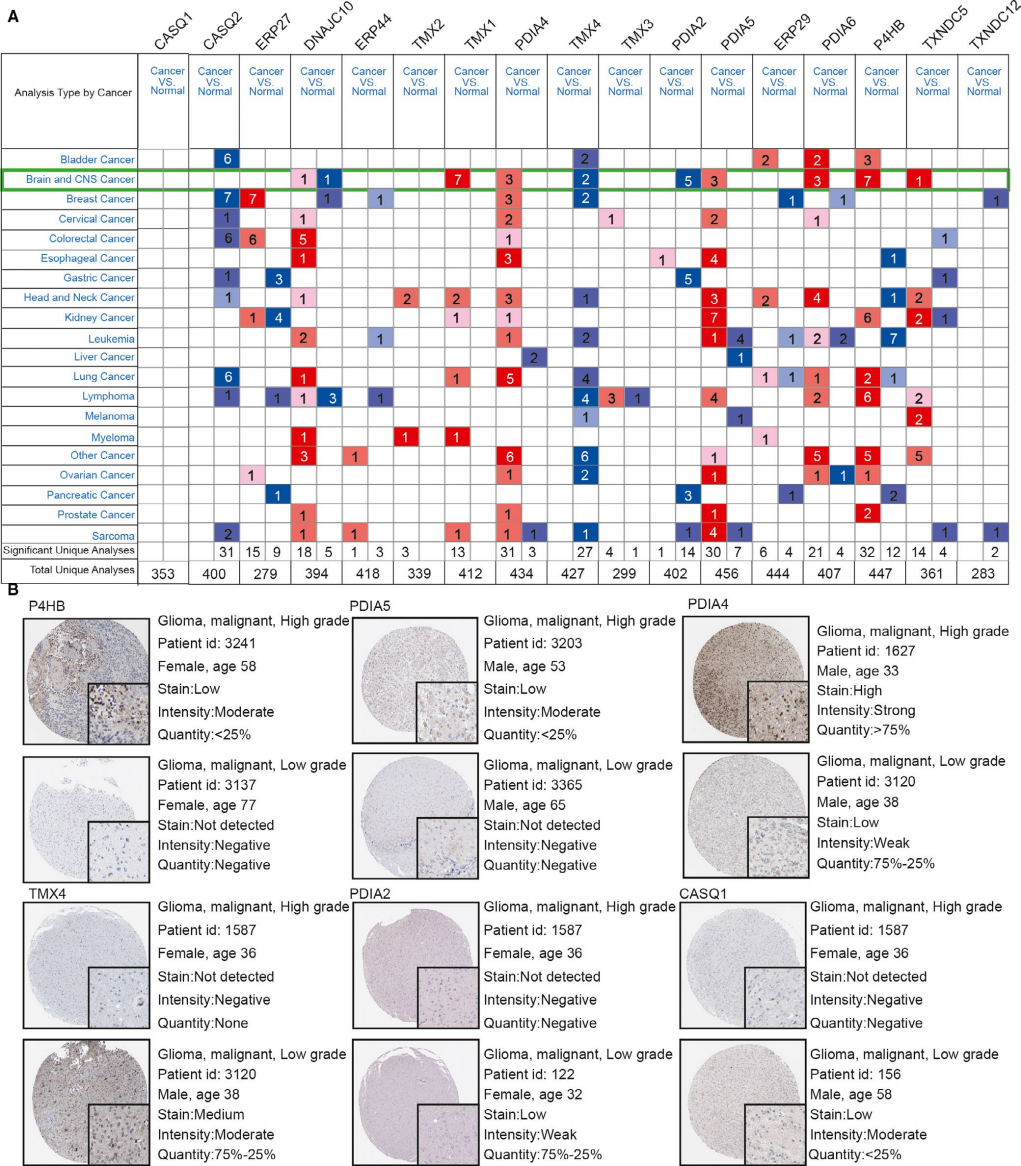
mRNA expression of PDI members in brain cancer samples identified by ONCOMINE data mining and the different PDI members in glioma tissues identified by immunohistochemistry images from the Human Protein Atlas. The differential expression of 17 PDI members was compared by the Student's t test (A). Thresholds were set as follows: *P*‐value: 1 × 10^−4^; fold change: 2; gene rank: 10%; data type: mRNA. The highest expression levels of *P4HB*,* PDIA5, TMX1*, *PDIA4*, *PDIA6* and *TXNDC5* were observed in high‐grade gliomas, whereas significant up‐regulation of *TMX4*, *PDIA2* and *CASQ1* was observed in low‐grade gliomas (B)

Changes in the transcript levels of the 17 PDI family members in different types of brain cancer and normal brain tissue were analysed by ONCOMINE data mining, while the relationship between PDI expression and different glioma pathological grades was analysed *via* the Human Protein Atlas database. ONCOMINE data mining indicated that the expression of *DNAJC10*, *TMX1*, *PDIA4*, *PDIA5*, *PDIA6*, *P4HB* and *TXNDC5* was significantly up‐regulated in brain and CNS cancers when compared with normal tissue; however, the expression levels of *TMX4* and *PDIA2* were higher in normal brain samples than in brain cancer tissues (Figure 2A). The Human Protein Atlas database was used to investigate the protein expression patterns of the 17 PDI family members in glioma. Immunohistochemistry staining data suggested that the expression of P4HB, PDIA5, TMX1, PDIA4, PDIA6, DNAJC10, TMX3, ERP44, ERP29, ERP27 and TXNDC5 was positively correlated with glioma grade (Figure 2B and Figure S1), whereas the expression levels of TMX4, PDIA2, TMX2 and CASQ1 were negatively correlated with glioma grade. No immunohistochemistry data were available for TXNDC12 and CASQ2 in the Human Protein Atlas database. Taken together, these results indicated that the data for the relationships between the expression of the 17 PDIs and the pathological grade of glioma extrapolated from the TCGA and CGGA data sets were reliable.

### 3.3 Stratification of the clinical outcomes and clinicopathological characteristics of gliomas based on consensus clustering analysis of the 17 PDI family members

**Figure 3:**
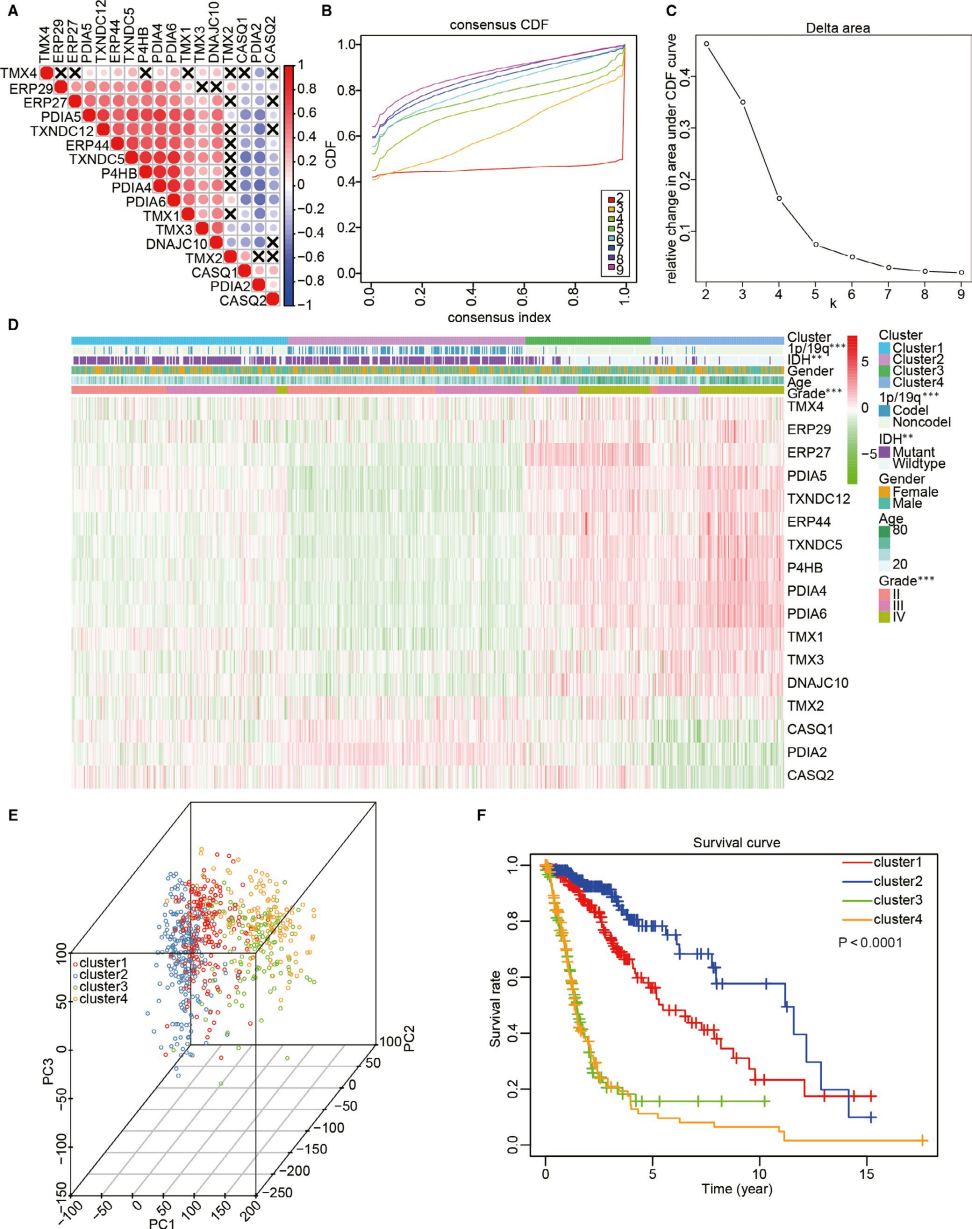
Comparison between clinicopathological features of gliomas and overall survival (OS) in four subgroups based on the differential expression of the 17 PDI family members in TCGA data set. (A) Spearman's correlation analysis of the 17 PDI members. (B) Consensus clustering cumulative distribution function (CDF) for *k* = 2‐10. (C) The delta area under the CDF curve for *k* = 2‐10. (D) Clinicopathological features of the four subgroups defined by the consensus expression of the 17 PDI family members. (E) Principal component analysis of all the RNA‐seq profiles in TCGA data set. (F) The 689 glioma samples in TCGA data set were analysed by Kaplan‐Meier OS curves

To investigate the relationship between the 17 PDIs, their RNA‐seq profiles were extracted from TCGA for correlation analysis. The results showed that a significant positive correlation existed between the expression of *ERP27*, *PDIA5*, *TXNDC12*, *ERP44*, *TXNDC5*, *P4HB*, *PDIA4*, *PDIA6*, *TMX1*, *TMX3* and *DNAJC10* in gliomas, whereas the expression of all these genes was negatively correlated with *CASQ1* and *PDIA2* (Figure 3A). Consistent with these results, the expression levels of ERP27, PDIA5, TXNDC12, ERP44, TXNDC5, P4HB, PDIA4, PDIA6, TMX1, TMX3 and DNAJC10 were shown to be positively correlated with the malignant progression of gliomas, while those of CASQ1 and PDIA2 were negatively correlated with increased malignancy of these tumours. We also used consensus clustering analysis to stratify TCGA of gliomas into four clusters with different clinical outcomes and clinicopathological characteristics based on the expression levels of the 17 members of the PDI family obtained from the TCGA data set. Based on the cumulative distribution function (CDF) curves of the consensus score and SigClust analysis (Figure 3B,C), *K* = 4 seemed to be an optimal selection with clustering stability increasing from *k* = 2‐10 in the TCGA data set, and the consensus score matrix of the PDIs mRNA expression in gliomas from TCGA data set were performed to consider the influence of correlation within and between groups (Figure S2). The result showed that the four clusters correlated with different clinicopathological features of gliomas (Figure 3D). We found that IDH‐mutant status, 1p/19q codeletion status and lower WHO grade of gliomas presented higher level in the cluster 2 compared with the other clusters (Table S2). In contrast, the cluster 4 was significantly correlated with wild‐type IDH status (*P* < .0001), 1p/19q non‐codeletion status (*P* < .0001) and higher WHO grade of gliomas (*P* < .0001) compared with the other clusters. PCA was used to compare transcripts between the four subgroups in TCGA (Figure 3E). The results indicated that significant differences existed between these subgroups. Furthermore, Kaplan‐Meier curves suggested that the OS in cluster 4 was significantly lower than in the other subgroups (*P* < .0001) based on the lower expression levels of *CASQ1*, *PDIA2* and *CASQ2*, and the higher expression levels of *DNAJC10*, *ERP29*, *TMX1*, *P4HB*, *PDIA4*, *PDIA5*, *PDIA6*, *TMX3* and *TXNDC5* (Figure 3F and Figure S3). Interestingly, cluster 2 exhibited lower expression levels of *DNAJC10*, *ERP29*, *TMX1*, *P4HB*, *PDIA4*, *PDIA5*, *PDIA6*, *TMX3* and *TXNDC5*, and the higher expression levels of *CASQ1*, *PDIA2* and *CASQ2*, coinciding with a significantly higher OS when compared with the other subgroups (*P* < .0001).

### 3.4 Consensus clustering analysis revealed the potential mechanisms underlying the malignant progression of gliomas

**Figure 4:**
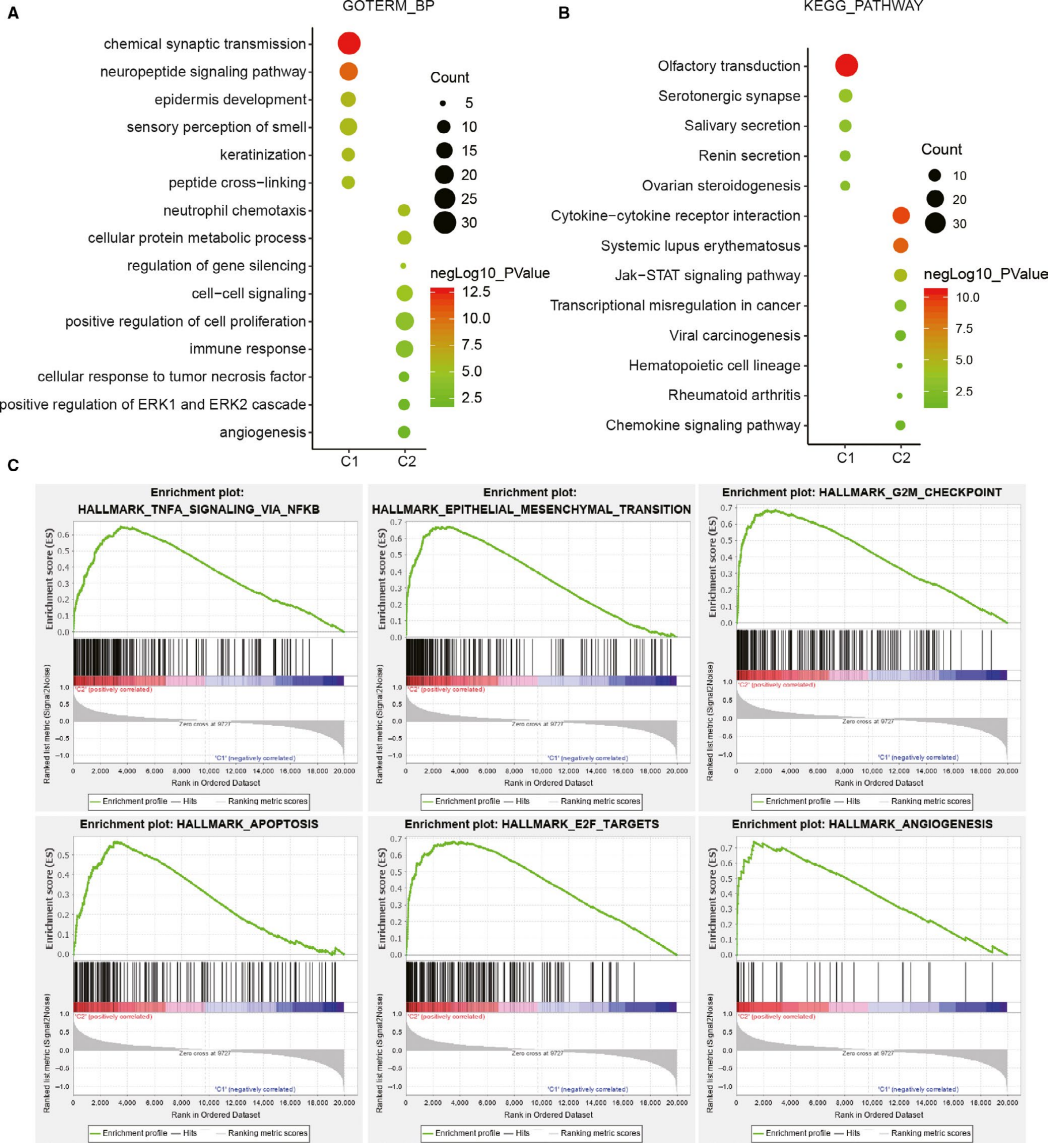
The potential biological functions of cluster 4 (C2) compared with those of the other clusters (C1). (A, B) Gene ontology (GO) biological process terms (A) and Kyoto Encyclopedia of Genes and Genomes (KEGG) pathway analysis (B) were utilized for the functional annotation of differentially expressed genes in C2 compared with C1. (C) Hallmarks of malignant tumours enriched in C2 revealed by gene set enrichment analysis (GSEA)

Because cluster 4 presented the lowest OS, the malignancy‐related mechanisms were further investigated in this cluster. All the samples were classified into two groups, C1 and C2, representing clusters 1/2/3 and cluster 4, respectively. We performed a differential gene expression analysis for all the transcriptional data, setting a *P* < .05 and log 2|fold change| >1. Compared with C1, gene functional enrichment analysis by DAVID (https://david.ncifcrf.gov/) revealed that malignancy‐related biological processes were enriched in C2 with its up‐regulated genes, including ‘neutrophil chemotaxis’, ‘cellular protein metabolic process’, ‘regulation of gene silencing’, ‘cell‐cell signalling’, ‘positive regulation of cell proliferation’, ‘immune response’, ‘cellular response to tumour necrosis factor’, ‘positive regulation of ERK1 and ERK2 cascade’ and ‘angiogenesis’, which were similarly consistent with the Kyoto Encyclopedia of Genes and Genomes (KEGG) pathway analysis (Figure 4A,B). In addition, GSEA also identified that hallmarks of malignant tumours, including TNFA signalling via NF‐κB (NES = 1.55, normalized *P* = .028), epithelial‐mesenchymal transition (NES = 1.58, normalized *P* = .006), G2M checkpoint (NES = 1.61, normalized *P* = .021), apoptosis (NES = 1.65, normalized *P* = .004), E2F targets (NES = 1.61, normalized *P* = .031) and angiogenesis (NES = 1.63, normalized *P* = .002), were significantly correlated with C2 (Figure 4C). In conclusion, these results indicated that the subgroups defined by consensus clustering exhibited a close relationship with the malignant progression of gliomas.

### 3.5 Prognostic value of the 17 PDI family members in gliomas and construction of a risk prognosis model based on 9 selected PDIs

**Figure 5:**
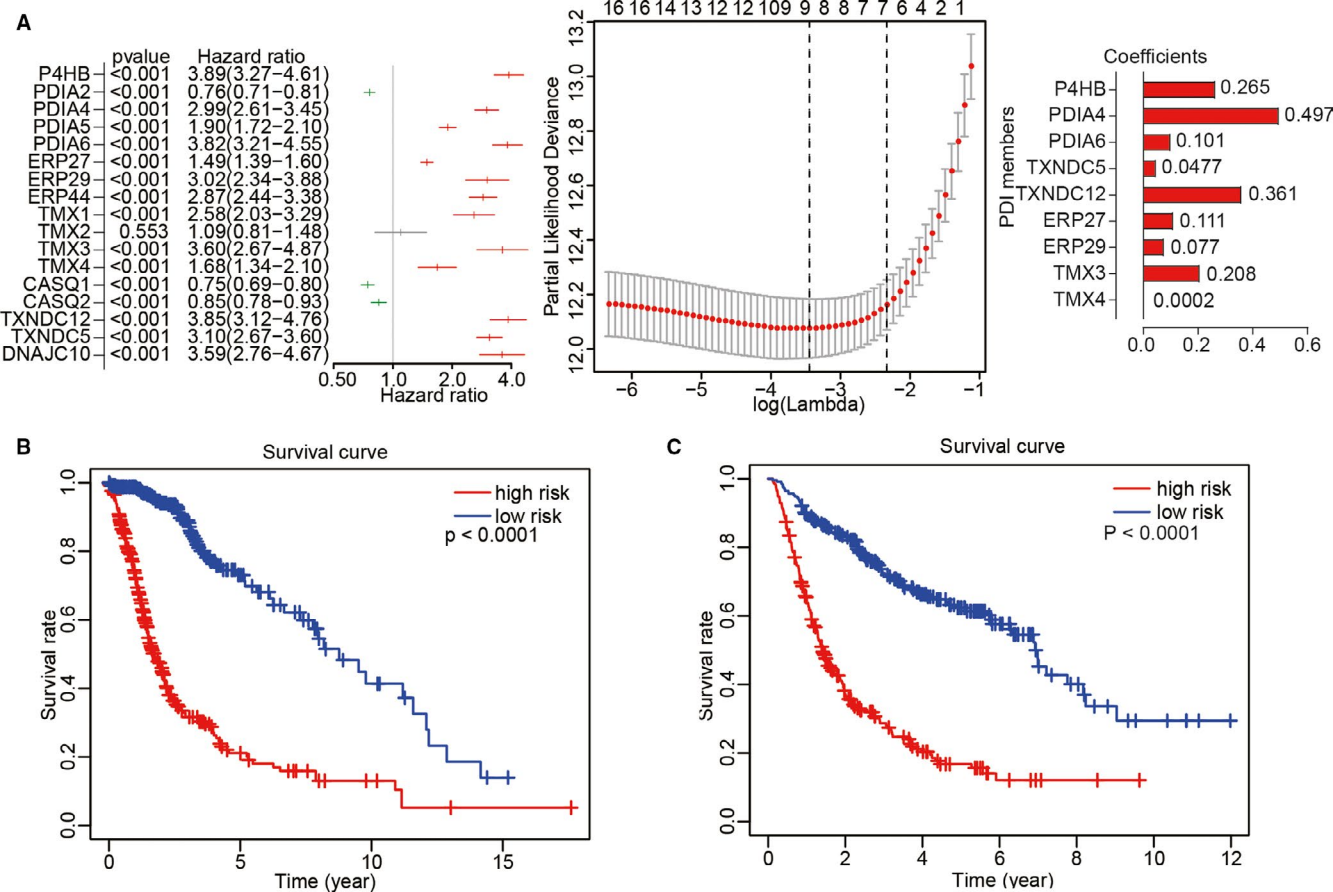
Construction of risk models and their prognostic characteristics. (A) Nine PDI family members were used to construct the risk models. Hazard ratios (HRs) and 95% confidence intervals (CIs) were calculated from univariate Cox regression analysis, and coefficients were calculated from multivariate Cox regression analysis using LASSO. (B, C) Survival analysis was performed using the Kaplan‐Meier method for the overall survival of patients in TCGA (B) and CGGA (C) data sets categorized into low‐ and high‐risk groups according to the risk score

Univariate Cox regression analysis was utilized to investigate whether the expression levels of the 17 PDI family members obtained from the TCGA were associated with glioma prognosis. The results indicated that these genes were significantly correlated with OS (*P* < .001), except for *TMX2*. Moreover, *PDIA2*, *CASQ1* and *CASQ2* were identified as protective genes with a hazard ratio (HR) <1. In contrast, *P4HB*, *PDIA4*, *PDIA5*, *PDIA6*, *ERP27*, *ERP29*, *ERP44*, *TMX1*, *TMX3*, *TMX4*, *TXNDC12*, *TNXDC5* and *DNAJC10* were identified as risk genes with a HR > 1. Furthermore, LASSO Cox regression analysis was performed to build an improved clinical prognosis model for gliomas based on the 17 PDI family members obtained from TCGA data set that were defined as the training set (Figure 5A). To investigate the prognostic value of the risk model based on nine genes selected by LASSO Cox regression analysis, TCGA (n = 689) and CGGA (n = 508) data sets were categorized into low‐ and high‐risk groups according to the median risk score. As shown in Figure 5B,C, Kaplan‐Meier survival analysis indicated that significant differences in OS existed between the two groups.

### 3.6 The prognostic scores of the risk model showed a strong correlation with the clinicopathological features of gliomas

**Figure 6:**
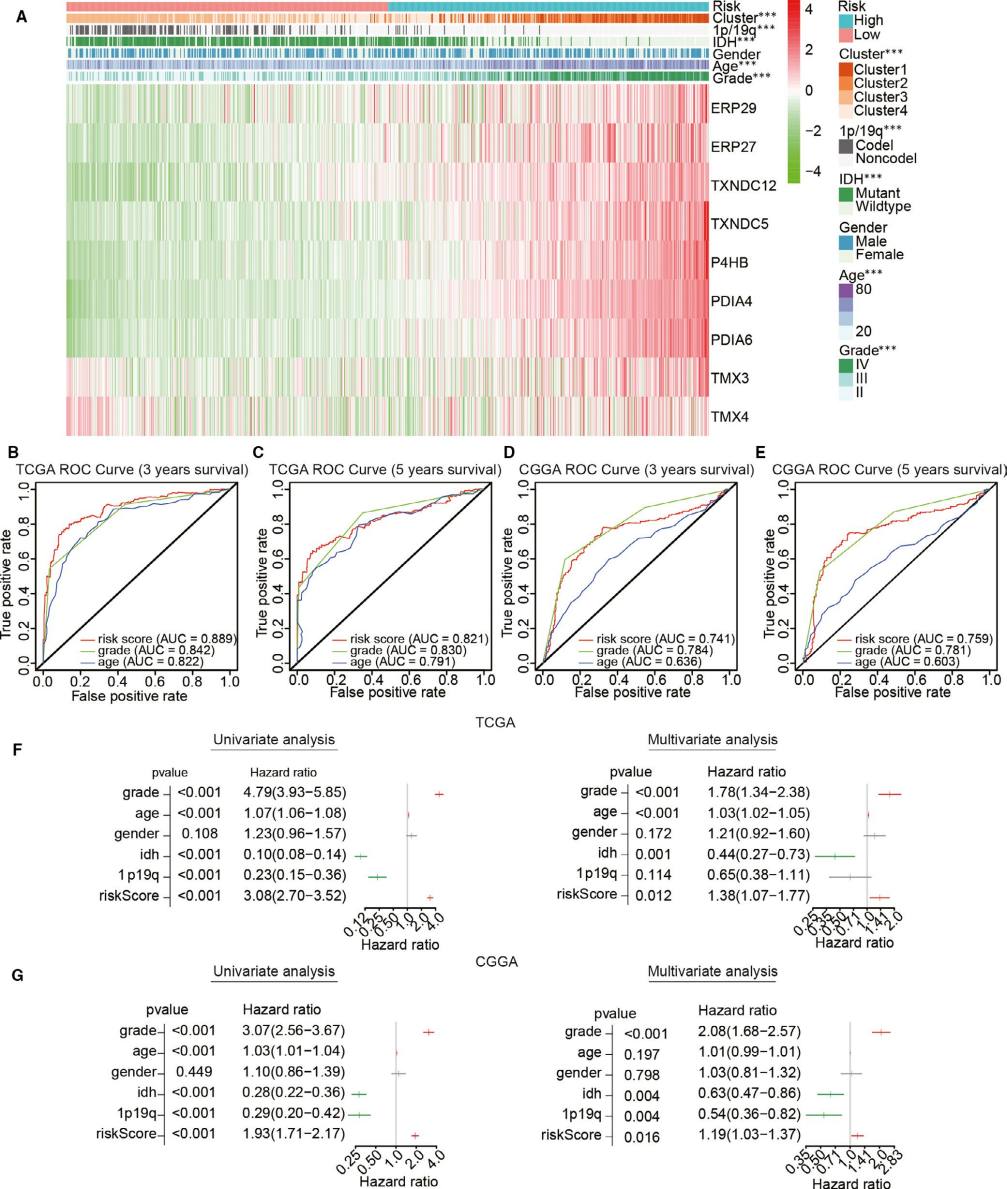
Relationship between risk score, clinicopathological features and clusters. (A) Heat map depicting the expression levels of the nine PDI family members in low‐ and high‐risk gliomas. The distribution of clinicopathological features was compared between the low‐ and high‐risk groups. ***P < .0001. (B–E) The predictive efficiency of the risk score, WHO grade and age in the training (TCGA) data set (B, C) and validation (CGGA) data set (D, E), shown by ROC curves based on 3‐ and 5‐year survival rates. (F, G) Univariate and multivariate Cox regression analyses of the association between clinicopathological factors, risk score and overall survival of patients in TCGA (F) and CGGA (G) data sets. ***P < .0001

The chi‐squared test was used to investigate the relationship between the prognostic risk scores and pathological characteristics of gliomas from the TCGA (Figure 6A). The result suggested that significant differences existed between clusters (*P* < .001), 1p/19q status (*P* < .001), IDH status (*P* < .001), age (*P* < .001) and WHO grade (*P* < .001; Table S3). Subsequently, the predictive value of the prognostic risk scores was evaluated by ROC curve analysis based on 3‐ and 5‐year survival rates. The area under the ROC curve (AUC) for OS was 0.889 at 3 years and 0.821 at 5 years, showing a reliable predictive ability in the CGGA validation data set (Figure 6B‐E). Overall, our study indicated that patient outcomes and clinicopathological features of glioma could be accurately predicted through the prognostic scores of the risk model.

To determine whether the risk scores could represent an independent prognostic index, we performed univariate and multivariate Cox regression analyses using TCGA data set. Univariate analysis showed that WHO grade, age, IDH status, 1p/19q status and risk score were closely correlated with OS (Figure 6F,G), while WHO grade (*P* < .001), age (*P* < .001), IDH (*P* = .001) and risk score (*P* = .012) remained a significant relationship with OS in the multivariate Cox regression analysis. The same analyses were also performed for the CCGA validation data set to analyse whether the risk score model constructed by the TCGA data set still has independent indicators. Multivariate regression analysis showed that WHO grade (*P* < .001), IDH status (*P* = .004), 1p/19q status (*P* = .004) and risk score (*P* = .016) were significantly associated with OS. Consequently, our results suggested that the prognostic value of PDIs in gliomas can be independently predicted by the risk score based on the nine selected PDI family members.

To investigate whether the prognostic value of the different WHO grades was significantly related to the risk signature, the Kaplan‐Meier method was used to compare the OS associated with different WHO grades between the low‐ and high‐risk group in the TCGA data set. The OS in the high‐risk group was shorter compared with that in the low‐risk group for WHO grade II and III gliomas (Figure S4A,B) and GBM (Figure S4C). These results were consistent with those for the CGGA validation data set, which showed a poor prognosis for the high‐risk group when compared with that in the low‐risk group for gliomas of differing WHO grades (Figure S4D‐F).

### 3.7 Analysis of the association between single nucleotide polymorphisms and tumour mutational burden with the different risk scores

**Figure 7:**
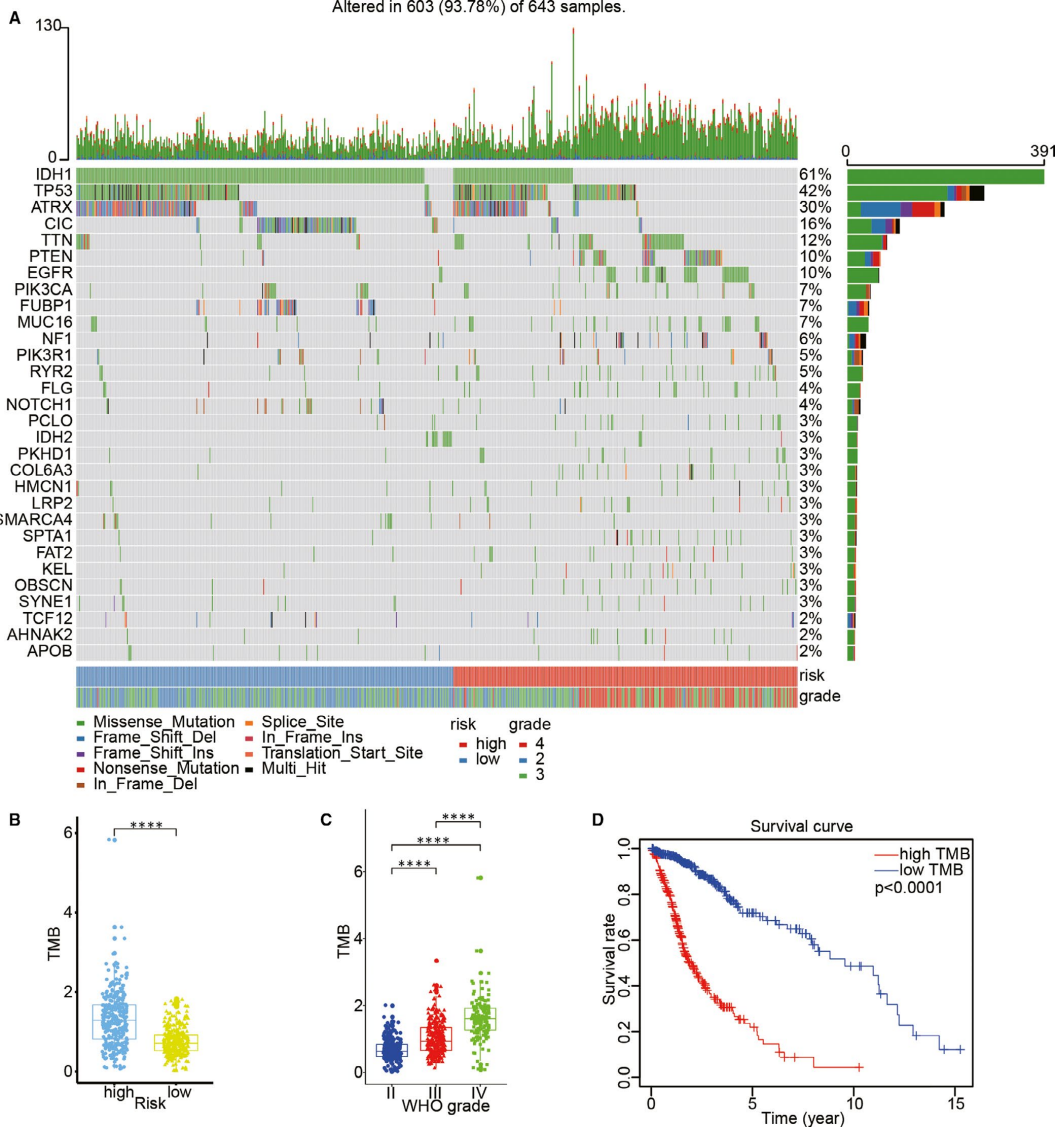
Association between risk score and mutational signature in glioma. (A) Genes with differentially SNPs associated with different risk scores. (B) The relationship between tumour mutation burden (TMB) and risk status. (C) The relationship between TMB and glioma WHO grade. (D) Survival analysis for the overall survival of the high‐TMB group and low‐TMB group using the Kaplan‐Meier method

The single nucleotide polymorphism (SNP) frequency in the TCGA data set was evaluated to determine the SNP status with different risk scores in GBM. The result suggested that SNPs in *IDH1* and *ATRX* exerted a higher somatic mutation burden in the low‐risk group than in the high‐risk group (Figure 7A). In contrast, the somatic mutation burden associated with *PTEN* and *EGFR* was higher in the high‐risk group than in the low‐risk group. Notably, the TMB was significantly different between the two groups (Figure 7B). The relationship between glioma WHO grade and TMB was also analysed (Figure 7C), and a positive correlation was found between the two. Subsequently, Kaplan‐Meier survival analysis suggested that OS with a high TMB was significantly shorter than that with a low TMB (Figure 7D).

## 4 DISCUSSION

Malignant glioma is the most common malignant primary central nervous system tumour in adults and is characterized by poor prognosis.^19^ The last few decades have witnessed the development of different treatments for glioma patients, such as precise surgical resection with pre‐operative imaging,^20^, ^21^ as well as radiation therapy^22^ and chemotherapy based on glioma histological identification.^23^ However, therapeutic targeting has always been necessary because of the short‐term survival of glioma patients after routine treatment.^23^, ^24^ Accumulating evidence has indicated that PDI has potential as a therapeutic target in cancer treatment.^13^ In addition, significantly elevated expression of PDI has been detected in various cancer types, including gliomas.^25^, ^26^ Therefore, in this study, we systematically analysed the potential function and prognostic value of PDI family members based on the different pathological features of gliomas.

Prolyl 4‐hydroxylase subunit beta (*P4HB*) encodes PDI (commonly referred to as PDIA1),^27^ a highly abundant ER‐resident protein that constitutes approximately 0.8% of the total cellular protein in mammals, and was also the first ER‐resident protein identified as a folding catalyst.^18^ Owing to the important role of PDI in cancer, *P4HB* is one of the most important genes in the PDI gene family. In this study, we analysed the correlation between the mRNA levels of 17 PDI family members and the malignancy of gliomas in TCGA and CGGA data sets. As expected, P4HB expression was positively correlated with increasing glioma WHO grade and was significantly up‐regulated in LGG samples with wild‐type IDH compared to LGG samples with mutant‐type IDH. Moreover, several studies have confirmed the cancer‐promoting effects of PDIA5 and TXNDC12 as important contributors to cancer progression and chemoresistance.^28^, ^29^ In this study, we have shown that the expression of *PDIA5* and *TXNDC12* was positively correlated with high‐grade gliomas, as well as with wild‐type IDH status and 1p/19q non‐codeletion. Interestingly, *CASQ1*, a gene related to myopathy,^30^ vacuolar myopathy^31^ and tubular aggregate myopathy,^32^ showed increased expression in samples of LGGs, as well as in those with mutated IDH and 1p/19q codeletion. However, no study has reported on the correlation between *CASQ1* and cancer, a topic that requires further study. Taken together, the results of our study have highlighted the close correlation existing between the differential expression of genes of the PDI family and the malignancy of gliomas.

Analyses based on the ONCOMINE and Human Protein Atlas databases suggested that the 17 PDI members identified in the TCGA data set have potential as diagnostic biomarkers in glioma. Consensus clustering analysis of the 17 PDI family members was utilized to classify the clinical outcomes and clinicopathological characteristics of glioma in the TCGA data set, resulting in the identification of four glioma subgroups. We found that cluster 4 was significantly correlated with poor prognosis as well as with wild‐type IDH status, 1p/19q non‐codeletion and higher WHO grade of gliomas. Our results also suggested that the PDI family was correlated with biological processes such as neutrophil chemotaxis, cellular protein metabolic process, positive regulation of cell proliferation, immune response, cellular response tumour necrosis factor and angiogenesis. Moreover, cancer‐related signalling pathways, such as transcriptional misregulation in cancer, the Jak‐STAT signalling pathway and viral carcinogenesis, were significantly correlated with PDI‐related gene expression. GSEA also identified hallmarks of malignant tumours, including apoptosis, E2F targets, epithelial‐mesenchymal transition, TNF signalling via NF‐κB and angiogenesis.

Taking the effect of the 17 PDI family members in the prognosis and malignant biological process of gliomas into account, we constructed a prognostic model with nine PDI members selected by LASSO Cox regression analysis to stratify glioma patients into high‐ and low‐risk categories based on the median risk score. Our result indicated that lower OS was significantly correlated with the high‐risk category in TCGA and CGGA data sets. Chi‐squared tests also highlighted that a strong correlation existed between the prognostic scores of the risk model and clinicopathological features of gliomas. In addition, our results suggested that the predictive value of the prognostic risk scores was robust based on ROC curve analysis, while univariate and multivariate Cox regression analysis revealed that the risk scores could be regarded as an independent prognostic index in TCGA and CGGA data sets. Subsequently, we used gliomas of differing WHO grades to evaluate the prognostic value of the risk signature in TCGA and CGGA data sets, and found that the prognostic value of the risk signature based on the nine selected PDI gene members was robust in WHO grade II, III or IV gliomas in both TCGA and CGGA data set.

Owing to the close relationship between SNPs and risk of tumour progression,^33^, ^34^, ^35^ we investigated the relationship between SNP and TMB status and the different risk scores. Mutational analysis of TCGA data set revealed that SNPs in *IDH1*, *ATRX*, *PTEN* and *EGFR* were significantly associated with risk status in glioma. Consistent with the fact that the mutation of *IDH1* and *ATRX* is associated with prolonged survival in glioma,^36^, ^37^ our result showed that the SNPs in *IDH1* and *ATRX* exerted a higher somatic mutation burden in the low‐risk group than in the high‐risk group. The loss of *PTEN* function was reported to promote tumour insensitivity to multiple therapeutic approaches, and the polymorphisms of *EGFR* were reported to increase the risk of gliomas^38^, ^39^, ^40^; similar results were found in our result that the SNPs with *PTEN* and *EGFR* were higher in the high‐risk group than in the low‐risk group. The TMB is an emerging biomarker for immunotherapy responses and the latest marker for the evaluation of PD‐1 antibody treatment efficacy in colorectal cancer. We found that TMB was higher in the high‐risk group than in the low‐risk group, and there was a positive correlation between TMB and glioma WHO grade. Kaplan‐Meier curve analysis further revealed that a high TMB was associated with a lower OS compared with that with a low TMB.

In summary, our study revealed the differential expression of the PDI gene family members in gliomas, as well as their potential function and prognostic values. These results may be useful for the development of clinically relevant treatments for gliomas.

## CONFLICT OF INTEREST

The authors declare that the research was conducted in the absence of any commercial or financial relationships that could be construed as a potential conflict of interest.

## AUTHOR CONTRIBUTIONS

Xingen Zhu designed the study; Qing hu and Chuming Tao performed the data analysis; Kai huang was responsible for the writing and critical reading of the manuscript; All authors read and approved the final manuscript.

## Supporting information

Fig S1

Fig S2

Fig S3

Fig S4

Table S1‐S3

Supplementary Material

## Data Availability

The data sets analysed for this study can be found in the TCGA (http://cancergenome.nih.gov/) and CGGA (http://www.cgga.org.cn/).
