# Supplementary figures and images for: Protein disulphide isomerase can predict the clinical prognostic value and contribute to malignant progression in gliomas
Source: J Cell Mol Med. 2020 Apr 17;24(10):5888–900. doi: 10.1111/jcmm.15264 (PMC7214159; doi:10.1111/jcmm.15264)

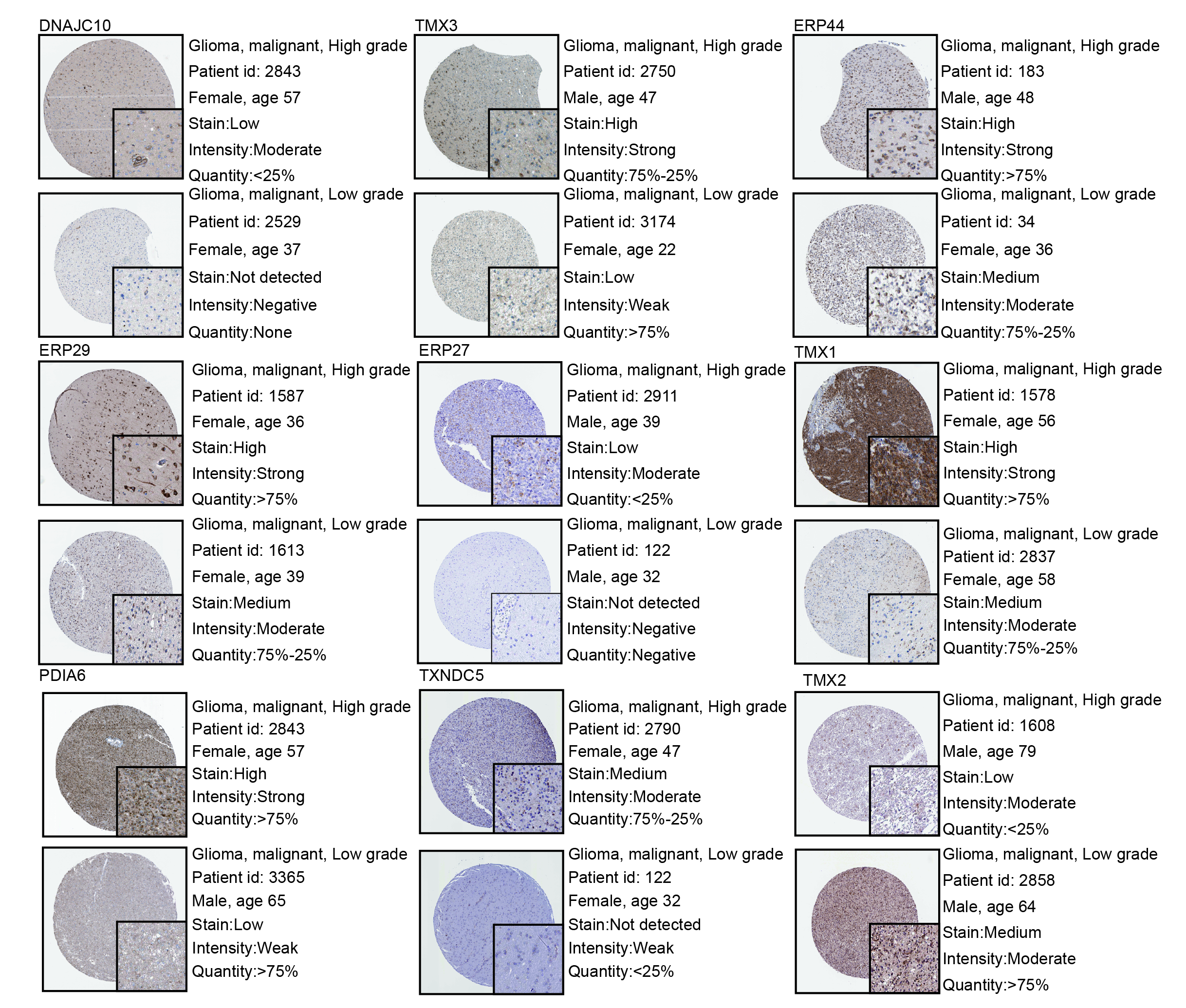

Supplement: Supplementary file 1 — Fig S1 [file JCMM-24-5888-s001.tif]

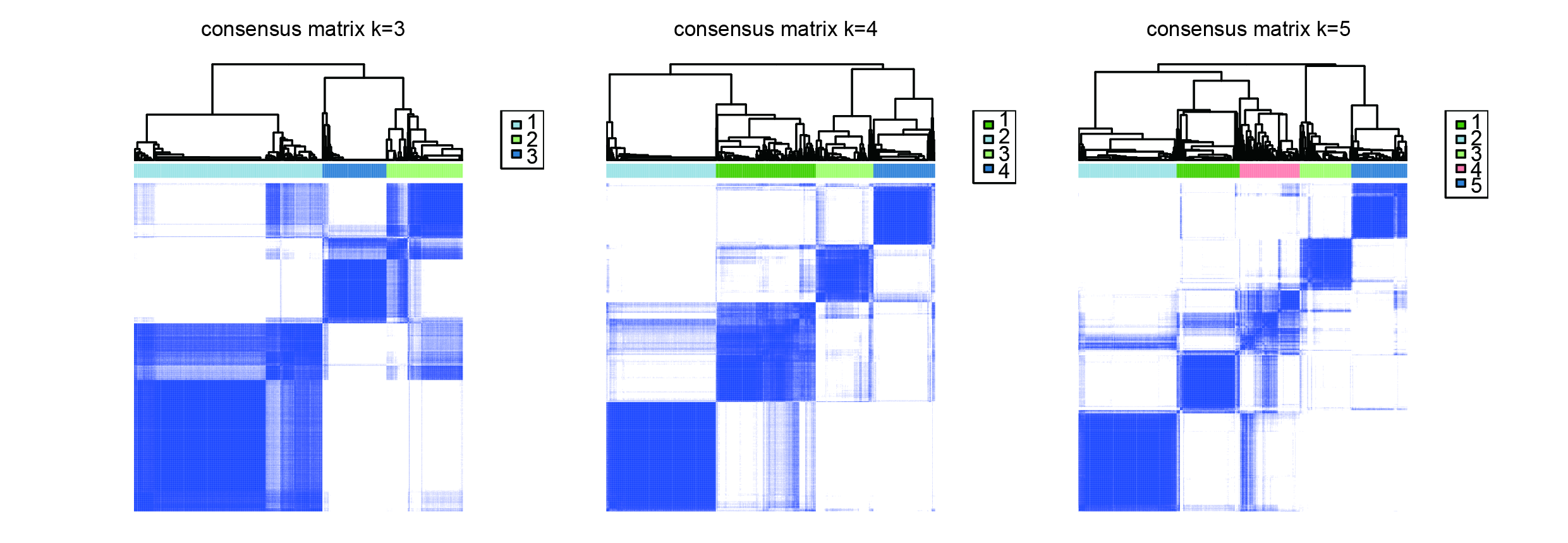

Supplement: Supplementary file 2 — Fig S2 [file JCMM-24-5888-s002.tif]

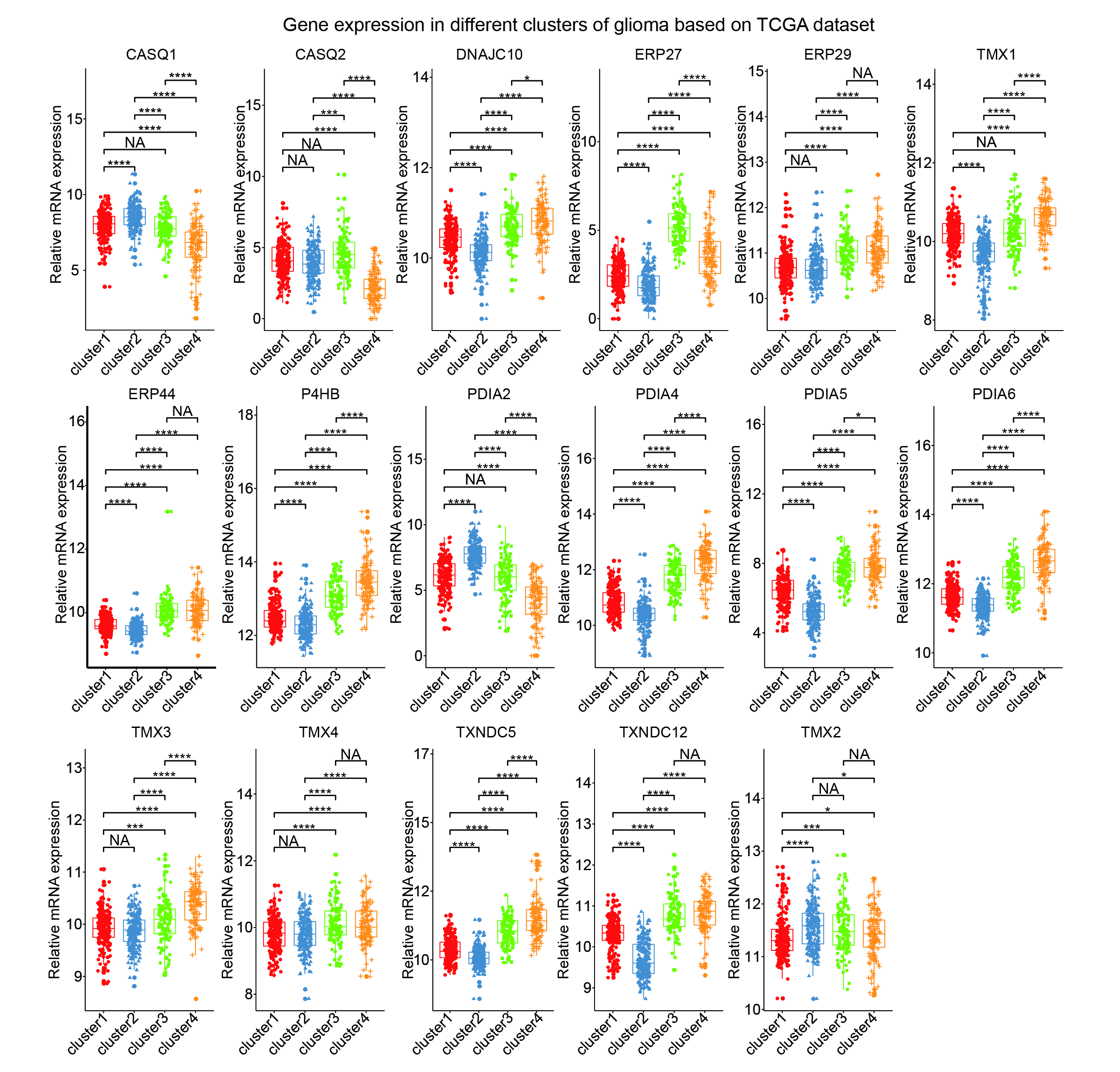

Supplement: Supplementary file 3 — Fig S3 [file JCMM-24-5888-s003.tif]

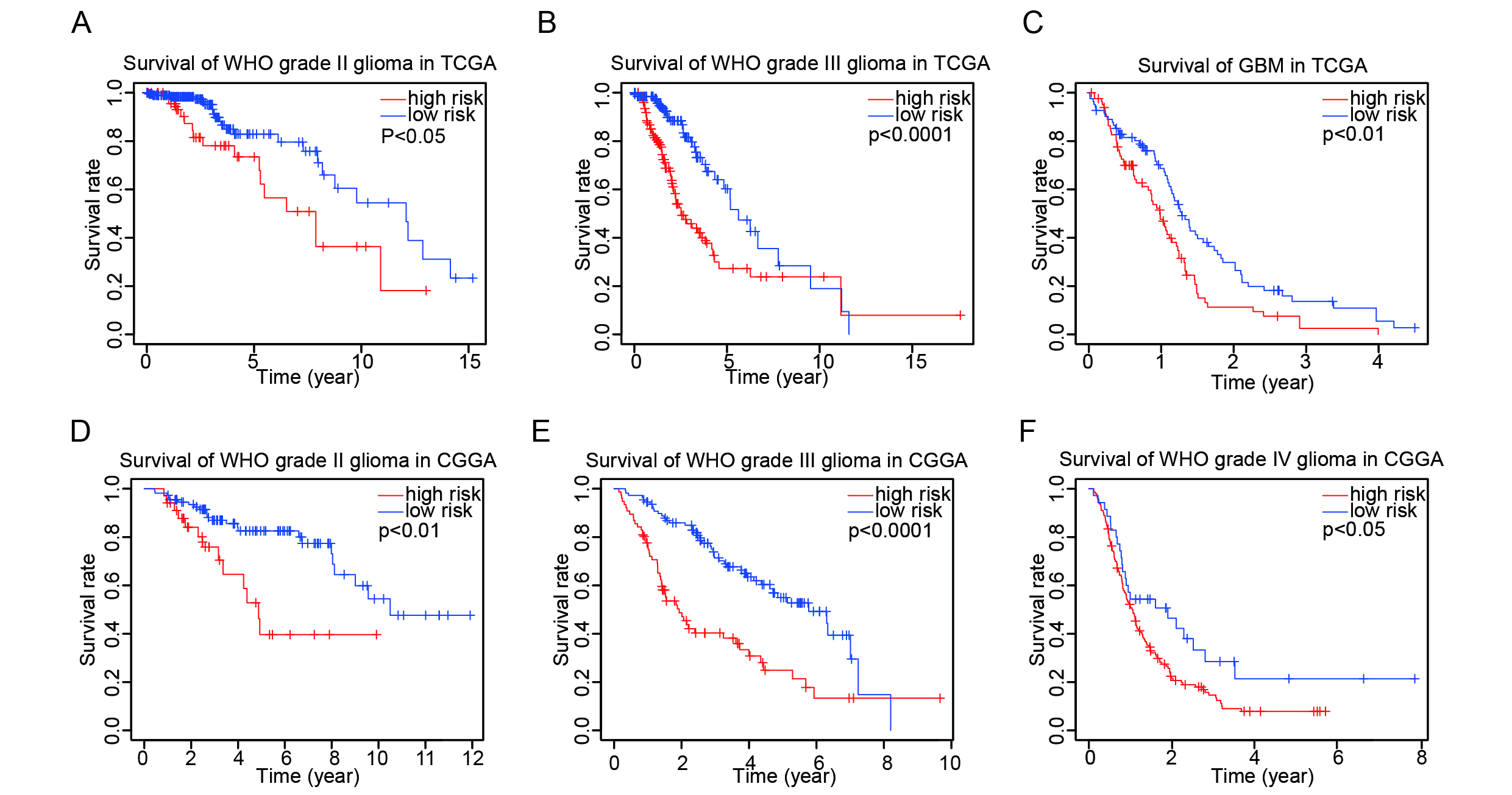

Supplement: Supplementary file 4 — Fig S4 [file JCMM-24-5888-s004.tif]
