# Supplementary material for: Protein disulphide isomerase can predict the clinical prognostic value and contribute to malignant progression in gliomas
Source: J Cell Mol Med. 2020 Apr 17;24(10):5888–900. doi: 10.1111/jcmm.15264 (PMC7214159; doi:10.1111/jcmm.15264)
Supplement: Supplementary file 5 — Table S1‐S3 [file JCMM-24-5888-s005.docx]

**Supplemental Information**

**Protein Disulfide Isomerase can Predict the Clinical Prognostic Value and Contribute to Malignant Progression in Gliomas**

Qing hu^1,2+^, Kai huang^1+^, Chuming Tao^1,2+^ and Xingen Zhu^1*^

^1^Department of Neurosurgery, The Second Affiliated Hospital of Nanchang University, Nanchang, Jiangxi 330006, P.R. China；

^2^East China Institute of Digital Medical Engineering, Shangrao, Jiangxi 334000, P.R. China.

***Correspondence:** Xingen Zhu, Department of Neurosurgery, The Second Affiliated Hospital of Nanchang University, Nanchang, Jiangxi 330006, P.R. China. Phone: +86-0791-86297662, E-mail: [zxg2008vip@163.com](mailto:zxg2008vip@163.com)

**Supplementary Tables**

**Table S1.** The clinicopathological information for the CGGA and TCGA datasets.

|  | | TCGA dataset | |  | CGGA dataset | |
| --- | --- | --- | --- | --- | --- | --- |
|  |  | Number | Percentage |  | Number | Percentage |
| Total |  | 689 | 100.00% |  | 508 | 100.00% |
| Age |  | 14-89 (46) |  |  | 11-73 (43) |  |
|  | <median | 342 | 49.64% | <median | 252 | 49.61% |
|  | ≥median | 347 | 50.36% | ≥median | 256 | 50.39% |
| Gender |  |  |  |  |  |  |
|  | Female | 295 | 42.82% | Female | 218 | 42.91% |
|  | Male | 394 | 57.18% | Male | 290 | 57.09% |
| Grade |  |  |  |  |  |  |
|  | II | 255 | 37.01% | II | 144 | 28.35% |
|  | III | 269 | 39.04% | III | 185 | 36.42% |
|  | IV | 165 | 23.95% | IV | 179 | 35.24% |
| IDH |  |  |  |  |  |  |
|  | Mutation | 382 | 55.44% | Mutation | 286 | 56.30% |
|  | Wildtype | 234 | 33.96% | Wildtype | 222 | 43.70% |
|  | NA | 73 | 10.60% | NA | 0 | 0.00% |
| 1p19q |  |  |  |  |  |  |
|  | Codel | 151 | 21.92% | Codel | 110 | 21.65% |
|  | Non-codel | 469 | 68.07% | Non-codel | 398 | 78.35% |
|  | NA | 69 | 10.01% | NA | 0 | 0.00% |

**Table S2.** Clinicopathological features of the clusters included in this study.

| TCGA dataset | | | | | | |
| --- | --- | --- | --- | --- | --- | --- |
|  |  | Cluster1 | Cluster2 | Cluster3 | Cluster4 | P-value |
| Total cases |  | 209 | 140 | 121 | 129 |  |
| Gender |  |  |  |  |  | 0.228 |
|  | Male | 122 | 126 | 78 | 68 |  |
|  | Female | 87 | 104 | 43 | 61 |  |
| Age |  |  |  |  |  | <0.0001 |
|  |  | <=46 (144) | <=46 (140) | <=46 (33) | <=46 (34) |  |
|  |  | >46 (65) | >46 (90) | >46 (88) | >46 (95) |  |
| Grade |  |  |  |  |  | <0.0001 |
|  | II | 92 | 143 | 13 | 7 |  |
|  | III | 106 | 85 | 38 | 40 |  |
|  | IV | 11 | 2 | 70 | 82 |  |
| IDH |  |  |  |  |  | <0.0001 |
|  | Mutation | 158 | 185 | 24 | 15 |  |
|  | Wildtype | 28 | 11 | 90 | 105 |  |
|  | NA | 23 | 34 | 7 | 9 |  |
| 1p19q |  |  |  |  |  | <0.0001 |
|  | Codel | 25 | 120 | 2 | 4 |  |
|  | Non-codel | 163 | 75 | 112 | 119 |  |
|  | NA | 21 | 35 | 7 | 6 |  |

**Table S3.** Clinicolpathological features are different between Low-risk and High-risk.

| TCGA dataset | | | | |
| --- | --- | --- | --- | --- |
|  |  | Risk-low | Risk-high | P-value |
| Total cases |  | 345 | 344 |  |
| Cluster |  |  |  | <0.0001 |
|  | Cluster1 | 123 | 86 |  |
|  | Cluster2 | 217 | 13 |  |
|  | Cluster3 | 2 | 119 |  |
|  | Cluster4 | 3 | 126 |  |
| Gender |  |  |  | 0.559 |
|  | Male | 193 | 201 |  |
|  | Female | 152 | 143 |  |
| Age |  |  |  | <0.0001 |
|  |  | <=46 (233) | <=46 (118) |  |
|  |  | >46 (112) | >46 (226) |  |
| Grade |  |  |  | <0.0001 |
|  | II | 202 | 53 |  |
|  | III | 140 | 129 |  |
|  | IV | 3 | 162 |  |
| IDH |  |  |  | <0.0001 |
|  | Mutation | 286 | 96 |  |
|  | Wildtype | 12 | 222 |  |
|  | NA | 47 | 26 |  |
| 1p19q |  |  |  | <0.0001 |
|  | Codel | 136 | 15 |  |
|  | Non-codel | 164 | 305 |  |
|  | NA | 45 | 24 |  |
