# Supplementary material for: Protein disulphide isomerase can predict the clinical prognostic value and contribute to malignant progression in gliomas
Source: J Cell Mol Med. 2020 Apr 17;24(10):5888–900. doi: 10.1111/jcmm.15264 (PMC7214159; doi:10.1111/jcmm.15264)
Supplement: Supplementary file 6 — Supplementary Material [file JCMM-24-5888-s006.docx]

**Supplemental Information**

**Protein Disulfide Isomerase can Predict the Clinical Prognostic Value and Contribute to Malignant Progression in Gliomas**

Qing hu^1,2+^, Kai huang^1+^, Chuming Tao^1,2+^ and Xingen Zhu^1*^

^1^Department of Neurosurgery, The Second Affiliated Hospital of Nanchang University, Nanchang, Jiangxi 330006, P.R. China；

^2^East China Institute of Digital Medical Engineering, Shangrao, Jiangxi 334000, P.R. China.

***Correspondence:** Xingen Zhu, Department of Neurosurgery, The Second Affiliated Hospital of Nanchang University, Nanchang, Jiangxi 330006, P.R. China. Phone: +86-0791-86297662, E-mail: [zxg2008vip@163.com](mailto:zxg2008vip@163.com)

**Supplementary Figures**

**Figure S1.** The protein expression patterns of the 17 PDI family members in glioma are different analyzed by Immunohistochemistry staining data in the Human Protein Atlas database.

**Figure S2. The consensus score matrix of the 17 PDI family genes mRNA expression in gliomas from TCGA.**

**Figure S3.** The mRNA expression patterns of the 17 PDI family members are different between different clusters of glioma according to the consensus clustering of the PDI gene family in TCGA dataset.

**Figure S4.** Analysis of the prognostic value of the risk signature in gliomas of differing WHO grades. (**A–C**) The overall survival (OS) of patients from TCGA dataset with different risk signatures in WHO grade II gliomas (**A**), WHO grade III gliomas (**B**), and glioblastoma multiforme (GBM) (**C**) analyzed by the Kaplan–Meier method. (**D**–**F**) Survival analysis for the OS of patients from the CGGA dataset with different risk signatures in WHO grade II gliomas (**D**), WHO grade III gliomas (**E**), and GBM (**F**) using the Kaplan–Meier method.
